# Moms Are Not OK: COVID-19 and Maternal Mental Health

**DOI:** 10.3389/fgwh.2020.00001

**Published:** 2020-06-19

**Authors:** Margie H. Davenport, Sarah Meyer, Victoria L. Meah, Morgan C. Strynadka, Rshmi Khurana

**Affiliations:** ^1^Program for Pregnancy and Postpartum Health, Faculty of Kinesiology, Sport, and Recreation, Women and Children's Health Research Institute, Alberta Diabetes Institute, University of Alberta, Edmonton, AB, Canada; ^2^Faculty of Medicine and Dentistry, Women and Children's Health Research Institute, University of Alberta, Edmonton, AB, Canada

**Keywords:** COVID-19, pregnancy, postpartum, mental health, physical activity

## Abstract

**Introduction:** Depression and anxiety affect one in seven women during the perinatal period, and are associated with increased risk of preterm delivery, reduced mother-infant bonding, and delays in cognitive/emotional development of the infant. With this survey we aimed to rapidly assess the influence of the COVID-19 pandemic and subsequent physical distancing/isolation measures on the mental health and physical activity of pregnant and postpartum women.

**Methods:** Between April 14 and May 8, 2020, we recruited women who were pregnant or within the first year after delivery to participate in an online survey. This included questionnaires on self-reported levels of depression/depressive symptoms (Edinburgh Postnatal Depression Survey; EPDS), anxiety (State-Trait Anxiety Inventory; STAI-State), and physical activity. Current and pre-pandemic values were assessed for each.

**Results:** Of 900 eligible women, 520 (58%) were pregnant and 380 (42%) were in the first year after delivery. Sixty-four percent of women reported reduced physical activity with the onset of isolation measures, while 15% increased, and 21% had no change to their physical activity. An EPDS score >13 (indicative of depression) was self-identified in 15% of respondents pre-pandemic and in 40.7% currently (mean ± SD; 7.5 ± 4.9 vs. 11.2 ± 6.3, respectively; *p* < 0.01, moderate effect). Moderate to high anxiety (STAI-state score >40) was identified in 29% of women before the pandemic (mean STAI = 34.5 ± 11.4) vs. 72% of women currently (mean STAI = 48.1 ± 13.6; *p* < 0.01, large effect). However, women engaging in at least 150 min of moderate intensity physical activity (meeting current guidelines) during the pandemic had significantly lower scores for both anxiety and depression than those who did not (*p* < 0.01, large and small effect, respectively).

**Discussion:** This rapid response survey identifies a substantial increase in the likelihood of maternal depression and anxiety during the COVID-19 pandemic. This highlights the strong need for heightened assessment and treatment of maternal mental health. However, these data also suggest that physical activity, which has previously been shown to reduce depression and depressive symptoms in pregnancy, may be associated with better mental health during the pandemic.

## Introduction

Since COVID-19 was first recognized in late 2019, the virus has rapidly spread throughout the world. In an effort to mitigate the devastating effects of this virus, varying levels of “stay at home” orders have been implemented in most countries around the world. This has resulted in the closure of schools, daycares, workplaces, and non-essential services. The impact of the physical (and social) isolation on mental health is anticipated to be high, and may disproportionately affect high risk populations.

Depression and anxiety affect one in seven women during the perinatal period, and are associated with increased risk of preterm delivery, reduced mother-infant bonding, and delays in cognitive/emotional development of the infant, which may persist into childhood (1–4). Prevention and treatment is critical yet it is estimated that 50% of women who are depressed remain undiagnosed during and following pregnancy (5). A cross-sectional study of 100 pregnant women from Italy found a moderate-to-severe psychological impact of the COVID-19 pandemic and highlighted the need for intervention to improve the mental health of this population (6). Furthermore, the COVID-19 pandemic is anticipated to decrease access to diagnosis and psychological or pharmacological treatment; this is likely exacerbating poor mental health (7). Even in the absence of clinical depression or anxiety, identifying therapies to reduce sub-clinical symptoms is important.

Obstetrical guidelines around the world recommend that all pregnant women without contraindication be physically active throughout pregnancy (8–11). This derives many health benefits including a 67% reduction in the odds of prenatal depression (odds ratio 0.33, 95% CI 0.21–0.53, *I*^2^ = 0%) (12), as well as postpartum depressive symptoms (standardized mean difference −0.34, 95% CI −0.50 to −0.19, *I*^2^ = 0%) (13). With this survey, we aimed to assess the influence of the COVID-19 pandemic and subsequent physical distancing/isolation measures on the mental health and physical activity of pregnant and postpartum women.

## Materials and Methods

This study was conducted in accordance with the Declaration of Helsinki, and was approved by the Ethics Committee at the University of Alberta (University of Alberta ethics protocol PRO00099671). Between April 14–May 8, 2020, we recruited women who were pregnant or within the first year after delivery to participate in an online survey. The survey was posted online via social media platforms (Twitter, Facebook, and Instagram) and shared publicly to facilitate snowball sampling. Participants were informed of the purpose, risks, and benefits of the survey, were told they could withdraw from the survey at any time, for any reason, and provided electronic informed consent. Women answered questions on demographics including their year of birth, level of education, and personal health history. They responded to questions regarding symptoms, testing, and diagnosis of COVID-19, and current physical distancing/isolation measures including current work status. Participants completed validated standard questionnaires of self-reported levels of depression/depressive symptoms (Edinburgh Postnatal Depression Survey; EPDS) and anxiety (State-Trait Anxiety Inventory; STAI-State). Self-reported physical activity was also collected. All measured were assessed for both current and pre-pandemic values.

The Edinburgh Postnatal Depression Scale (EPDS) is a self-reported screening questionnaire consisting of 10 questions which was initially used in the postnatal period; however, it is also commonly used during pregnancy (14). Clinical diagnosis of depression can only be determined by a trained health professional; however, a score of >13 on the EPDS is associated with a likely diagnosis of depression. The State-Trait Anxiety Inventory (STAI) is a commonly used self-report questionnaire to screen for the presence and severity of state (i.e., right now) and trait (how prone a person is to anxiety) anxiety (used with permission) (15). The STAI consists of 40 questions with equal numbers assessing both the state and trait subscales. A score of 40 or higher has been identified as the threshold to identify clinically significant symptoms of anxiety (16).

Physical activity was self-reported in two ways. First, participants provided an overall assessment of their achievement of 150 min of moderate intensity physical activity each week (i.e., current recommendations for pregnant and postpartum women). Secondly, participants reported on physical activity during the week. Volume of physical activity was determined as per previously published methods (12, 17). The intensity of each activity was assigned a metabolic equivalents (METs) score using the Compendium, and multiplied by the frequency and duration of the activities (18).

All data were checked for accuracy, and invalid data were removed. Pre-pandemic versus current mental health and physical activity metrics were compared using paired *t*-tests or Kruskal-Wallis-H tests as appropriate according to the normality of their distribution. Effect size was determined using Cohen's *d*. Women were stratified based on physical activity pattern during the pandemic to assess its influence on mental health using ANOVA. *Post-hoc* comparisons were assessed using Dunns Method. Statistical significance was defined as *p* < 0.05 and analyzed using SigmaStat (Systat Software Inc., USA).

## Results

Of 900 eligible women, 520 (58%) were pregnant and 380 (42%) were in the first year after delivery. One invalid record was removed. Participant's median age was 33 years (range 17–49 years; *n* = 862), 75.5% lived in cities (*n* = 651), and 69% (*n* = 595) lived in a single family home with an average of one child (range 0–5) living with them in the household. Most women were from North America (*n* = 779), were Caucasian (*n* = 736, Table 1), and had some postsecondary education (*n* = 520). At the time of the survey, 2.8% and 6.7% of women had a pre-existing clinical diagnosis of depression and anxiety, respectively (Table 1). Forty seven women had experienced symptoms of COVID-19, 13 of whom were tested and all had negative results. Ninety-three percent of women were currently engaged in physical distancing measures with 83% of women in self-isolation or isolation at home. Sixty-four percent of women reported reduced physical activity with the onset of isolation measures, while 15% increased and 21% had no change to their physical activity. The number of women meeting current prenatal physical activity recommendations prior to and during the pandemic are shown in Table 2.

**Table 1:** Participant characteristics.

	**Number (% out of 900)**
Ethnic background	
Caucasian	736 (81.8%)
Mixed Heritage	42 (4.7%)
Asian	36 (4%)
Hispanic or Latina	11 (1.2%)
African American	10 (1.1%)
Indigenous people	9 (1%)
South Asian	9 (1%)
Prefer not to say	58 (5.2%)
Region of residence	
Canada	655 (72.8%)
United Kingdom	73 (8.1%)
USA	53 (5.9%)
Australia	10 (1.1%)
India	7 (0.8%)
Brazil	6 (0.7%)
Germany	5 (0.6%)
China	4 (0.4%)
France	3 (0.3%)
Other/prefer not to say	84 (9.3%)
Relationship Status	
In a relationship but living together	837 (93%)
Single	21 (2.3%)
In a relationship but living apart	5 (0.6%)
Prefer not to say	37 (4.1%)
Employment status	No % due to multiple selections
Student	31
Self-employed	74
Part-time employment	89
Full-time employment	506
Homemaker/full time parent	103
Unemployed before COVID-19	16
Unemployed due to COVID 19	71
Prefer not to say	63
Pregnancy complications	
Gestational diabetes	37 (4.1%)
Hypertensive disorders of pregnancy	41 (4.6%)
Placenta previa	16 (1.8%)
Preterm labor	27 (3%)
Intrauterine growth restriction	11 (1.2%)
Multiple pregnancy (twins or higher)	22 (2.4%)
Depression	25 (2.8%)
Anxiety	60 (6.7%)
Prefer not to say	55 (6.1%)
No complications	655 (72.8%)
Pre-existing conditions	
Type 1 diabetes	5 (0.6%)
Type 2 diabetes	4 (0.4%)
Cardiovascular disease	6 (0.7%)
Respiratory disease	47 (5.2%)

**Table 2:** Self-reported physical activity pre-pandemic and following the implementation of governmental recommendations for self-isolation/physical distancing associated with the COVID-19 pandemic.

**Did you meet or exceed 150 min of moderate intensity physical activity each week?**	***N* (%) Total = 714**	**METs per week**
**Prior to the implementation of physical isolation measures of COVID-19:**		
Yes, most if not all of the time	205 (28.7%)	1,548 (1,120–2,342)
Yes, sometimes	211 (29.6%)	894 (567–1,372)^*^
Yes, but rarely	127 (17.8%)	580 (270–1,107)^*^^#^
No	171 (23.9%)	180 (0–516)^*^^#^^†^
**Following implementation of physical isolation measures of COVID-19**		
Yes, most if not all of the time	168 (23.5%)	1539 (967–2,301)
Yes, sometimes	195 (27.3%)	1005 (612–1,342)^*^
Yes, but rarely	136 (19%)	389 (180–767)^*^^#^
No	215 (30.1%)	90 (0–393)^*^^#^^†^

*METs, metabolic equivalents*.

*Main effect of group on METs pre-pandemic: H = 260.206 with 3 degrees of freedom, p < 0.001, Cohen's d 1.507, very large*.

*Main effect of group on METs current: H = 342.357 with 3 degrees of freedom, p < 0.001, Cohen's d 1.914, very large*.

* *Different from most, if not all of the time, p < 0.05*.

# *Different from sometimes, p < 0.05*.

† *Different from rarely, p < 0.05*.

An EPDS score >13 (indicative of depression) was self-identified in 15% respondents pre-pandemic and in 40.7% currently (mean ± SD; 7.5 ± 4.9 vs. 11.2 ± 6.3, respectively; *p* < 0.01, Cohen's *d* 0.66; moderate effect). Moderate to high anxiety (STAI-state score >40) was identified in 29% of women before the pandemic (mean STAI = 34.5 ± 11.4) vs. 72% of women currently (mean STAI = 48.1 ± 13.6; *p* < 0.01, Cohen's *d* 1.08; large effect). However, women engaging in at least 150 min of moderate intensity physical activity (meeting current guidelines) during the pandemic had significantly lower scores for both anxiety (large effect) and depression (small effect) than those who did not (*p* < 0.01, see Table 3).

**Table 3:** Current self-reported adherence to physical activity guidelines of at least 150 min of moderate to vigorous physical activity each week following the implementation of governmental recommendations for self-isolation/physical distancing associated with the COVID-19 pandemic.

**Meets or exceeds physical activity guidelines**	**Edinburgh Postnatal Depression Score (EPDS) Median (25, 75%)**	**State-Trait Anxiety Inventory (STAI-State) Median (25, 75%)**
Yes, most if not all of the time	8 (4, 14)	43 (32, 52)
Yes, sometimes	10 (5, 14)	47 (36, 56)^*^
Yes, but rarely	11 (8, 17)^*^	52 (42, 60)^*^
No	13 (8, 19)^*^^#^	53 (42, 62)^*^^#^

*Main effect of group on EPDS: H = 36.900 with 3 degrees of freedom, p < 0.001, Cohen's d = 0.491, small*.

*Main effect of group on STAI-State: H = 47.415 with 3 degrees of freedom p < 0.001, Cohen's d = 0.568, large*.

* *Different from most, if not all of the time, p < 0.05*.

# *Different from sometimes, p < 0.05*.

## Discussion

The findings of this survey illustrated a significant increase in self-reported levels of depression and anxiety, and substantial reductions in physical activity in pregnant women from before to during the COVID-19 pandemic. Depression and anxiety are well-established to have both acute (e.g., preterm delivery, attenuated fetal/neonatal growth) and long-term consequences (e.g., increased risk of future anxiety and depression, cognitive delays for the offspring) for the psychological and physical health of both mother and baby (2–4). Although, clinical diagnosis and treatment via psychological or pharmacological treatment remain front line therapies, the COVID-19 pandemic may reduce access and/or attendance to health care visits which could increase the risk of maternal/fetal health complications. The findings of this survey suggest that remaining physically active could be a helpful tool for pregnant and postpartum women. Specifically, engaging in at least 150 min of moderate intensity physical activity each week was associated with lower scores on screening tools for depression or anxiety. Thus, physical activity is an accessible measure to blunt the mental health crisis currently being experienced by pregnant and postpartum women.

Although estimates vary, depression and/or anxiety affect ~14% of pregnant and postpartum women (1). The consequences of undiagnosed and untreated depression are serious; nearly 20% of women with postpartum depression have considered hurting themselves and in the UK, the leading cause of maternal death in the year following delivery is suicide (19). Treatment of depression and anxiety is critical to support the health of both mother and child. However, many women are reluctant to take antidepressants even when prescribed (20, 21). In non-pregnant populations exercise has been found to be as effective in treating mild-to-moderate depression as anti-depressants and psychotherapy (22). Although this has not been evaluated in pregnant or postpartum women, recent systematic reviews and meta-analyses of randomized controlled trials have shown pre- and post-natal exercise reduces the odds of depression and depressive symptoms. The findings from the current study also suggest that pregnant or postpartum women who were able to engage in regular physical activity during the COVID-19 pandemic may have improved mental health compared to those who were not. We must also consider that certain barriers to physical activity may be increased in conjunction with COVID-19, such as the closure of indoor recreation centers and outdoor parks/greenspace. However, activities such as gardening, going for walks, household chores, and online fitness classes are feasible alternatives to promote wellness through movement and should be promoted as reasonable methods for increasing the physical activity of moms.

Due to the rapid development of COVID-19, pre-pandemic data were obtained through recall and were cross-sectional in nature, thereby precluding the ability to make causal inferences. As these data are correlative the underlying reason for the observed relationships cannot be determined and only associations could be identified. Indeed, a number of external factors may influence both likelihood of depression/anxiety and physical activity participation. These include fear of the virus, financial stresses, increased domestic workload, lack of motivation to exercise and social isolation, among many others. However, previous systematic reviews and meta-analyses from randomized controlled trials have demonstrated that rates of depression and depressive symptoms are reduced in pregnant and postpartum women randomized to an exercise intervention (compared to no exercise) (12, 13) supporting the observed relationship in the current study. Our approach utilized established and validated measures of self-reported screening tools for anxiety, depression, and physical activity to assess the psychological health of pregnant and postpartum women. These data were collected via online survey with social media as the primary avenue for promotion. As such, random sampling did not occur which may have introduced sampling bias into the survey. The number of individuals who saw the survey and chose not to participate could not be determined; however, it is plausible that women who had a pre-existing interest in physical activity and/or mental health would be more likely to respond to the survey. Furthermore, previous research has suggested that the quality of response may be reduced in online surveys (23, 24). Careless responding occurs when a participant fails to read or interpret the survey appropriately leading to incorrect responses. These types of responses can directly influence the results, thus the findings of this survey should be interpreted with consideration of these limitations. Our population was primarily from Canada (with a freely accessible health care system), Caucasian, were married, living in a single-family home, and had some post-secondary education. While we did not capture a more diverse population, the high rates of anxiety and depression are concerning as this group would not typically be considered at elevated risk of mental health disorders. Thus, these data likely under-estimate the true mental health crisis for pregnant and postpartum women as a result of the COVID-19 pandemic. Although the change in prevalence and symptom severity of anxiety and depression from pre-pandemic to current times may be subject to recall bias, the unexpectedly high rates of current mental health issues warrant an urgent call to action.

## Conclusion

This rapid response survey identifies a substantial increase in self-reported maternal depression and anxiety from pre- to during-pandemic. These data highlight the strong need for heightened assessment and treatment of maternal mental health. However, these data also suggest that remaining active during the pandemic is associated with a reduced likelihood of anxiety and depression. These data highlight a potential intervention for all pregnant and postpartum women to improve or maintain mental health during this extremely stressful period where access to diagnosis and treatment is more challenging.

## Data Availability Statement

The raw data supporting the conclusions of this article will be made available by the authors, without undue reservation.

## Ethics Statement

The studies involving human participants were reviewed and approved by Ethics Committee at the University of Alberta (University of Alberta ethics protocol PRO00099671). The patients/participants provided their written informed consent to participate in this study.

## Author Contributions

MD: had full access to all of the data in the study and takes responsibility for the integrity of the data, the accuracy of the data analysis, statistical analysis, and study supervision. MD and RK: study concept and design. All authors: acquisition, analysis, or interpretation of data, drafting of the manuscript, and critical revision of the manuscript for important intellectual content.

## Conflict of Interest

The authors declare that the research was conducted in the absence of any commercial or financial relationships that could be construed as a potential conflict of interest.
